# From Songlines to genomes: Prehistoric assisted migration of a rain forest tree by Australian Aboriginal people

**DOI:** 10.1371/journal.pone.0186663

**Published:** 2017-11-08

**Authors:** Maurizio Rossetto, Emilie J. Ens, Thijs Honings, Peter D. Wilson, Jia-Yee S. Yap, Oliver Costello, Erich R. Round, Claire Bowern

**Affiliations:** 1 National Herbarium of NSW, Royal Botanic Gardens and Domain Trust, Sydney, New South Wales, Australia; 2 Queensland Alliance of Agriculture and Food Innovation, University of Queensland, Brisbane, Australia; 3 Department of Environmental Sciences, Macquarie University, Sydney, New South Wales, Australia; 4 Biological Sciences, Leiden University, Sylviusweg, Leiden, the Netherlands; 5 Aboriginal Heritage and Joint Management Team, Office of Environment and Heritage, New South Wales, Australia; 6 Ancient Language Lab, School of Languages and Cultures, University of Queensland, Brisbane, Australia; 7 Department of Linguistics, Yale University, New Haven, Connecticut, United States of America; Indian Institute of Science, INDIA

## Abstract

**Background:**

Prehistoric human activities have contributed to the dispersal of many culturally important plants. The study of these traditional interactions can alter the way we perceive the natural distribution and dynamics of species and communities. Comprehensive research on native crops combining evolutionary and anthropological data is revealing how ancient human populations influenced their distribution. Although traditional diets also included a suite of non-cultivated plants that in some cases necessitated the development of culturally important technical advances such as the treatment of toxic seed, empirical evidence for their deliberate dispersal by prehistoric peoples remains limited. Here we integrate historic and biocultural research involving Aboriginal people, with chloroplast and nuclear genomic data to demonstrate Aboriginal-mediated dispersal of a non-cultivated rainforest tree.

**Results:**

We assembled new anthropological evidence of use and deliberate dispersal of *Castanospermum australe* (Fabaceae), a non-cultivated culturally important riparian tree that produces toxic but highly nutritious water-dispersed seed. We validated cultural evidence of recent human-mediated dispersal by revealing genomic homogeneity across extensively dissected habitat, multiple catchments and uneven topography in the southern range of this species. We excluded the potential contribution of other dispersal mechanisms based on the absence of suitable vectors and current distributional patterns at higher elevations and away from water courses, and by analyzing a comparative sample from northern Australia.

**Conclusions:**

Innovative studies integrating evolutionary and anthropological data will continue to reveal the unexpected impact that prehistoric people have had on current vegetation patterns. A better understanding of how traditional practices shaped species’ distribution and assembly will directly inform cultural heritage management strategies, challenge “natural” species distribution assumptions, and provide innovative baseline data for pro-active biodiversity management.

## Introduction

Studies of prehistoric human influences on the Australian vegetation have primarily centered around broad-scale change associated with the practice and cessation of Aboriginal burning [1], [2] and the hypothesized cumulative effects of human-induced decline of megafauna [3]. Within a broader context, the role of humans has been considered as an integral factor in the geographical spread and evolution of crops and agriculture. Evidence from Amazonian forests suggests that pre-Columbian dispersal of domesticated plants had an unforeseen role on how species are distributed in present-day rainforest assemblages [4], [5], [6]. In the Pacific region, DNA-based evolutionary studies have documented the link between long-distance seed dispersal by Indigenous people and the current distribution of native crops [7], [8], [9]. By integrating genetics, linguistics, and archaeobotany researchers have been able to investigate the prehistoric human settlement of the Pacific through the evolutionary history of traditional crop domestication [10]. Evidence regarding dispersal of non-cultivated plants by prehistoric Indigenous peoples however, remains limited to ecological and archaeological circumstantial evidence [11], [12]. The advent of new technological advances and the integration of multi-disciplinary data sources have the potential to innovate in the field.

In Australia, the influence of assisted migration or human dispersal has been included in lists of possible explanations for the landscape genetic patterns detected for *Livistona mariae* in central Australia [13] and *Adansonia gregorii* in the Kimberley region [14]. In the absence of direct archaeological evidence, the corroboration of human-mediated dispersal hypotheses and the exclusion of alternative mechanisms, requires for further ethnographic and cultural research to be directly linked to interpretations derived from genetic patterns. For instance, a follow-up study on *A*. *gregorii* identified overlap between population-level genetic connectivity and regional linguistic variation [15], and traditional local beliefs of Indigenous dispersal were reported within historical accounts related to the current distribution of *L*. *mariae* [16]. However, these interpretative frameworks lack corroboration from other sources and ethnographic information directly derived from Indigenous collaborations.

**Fig 1:**
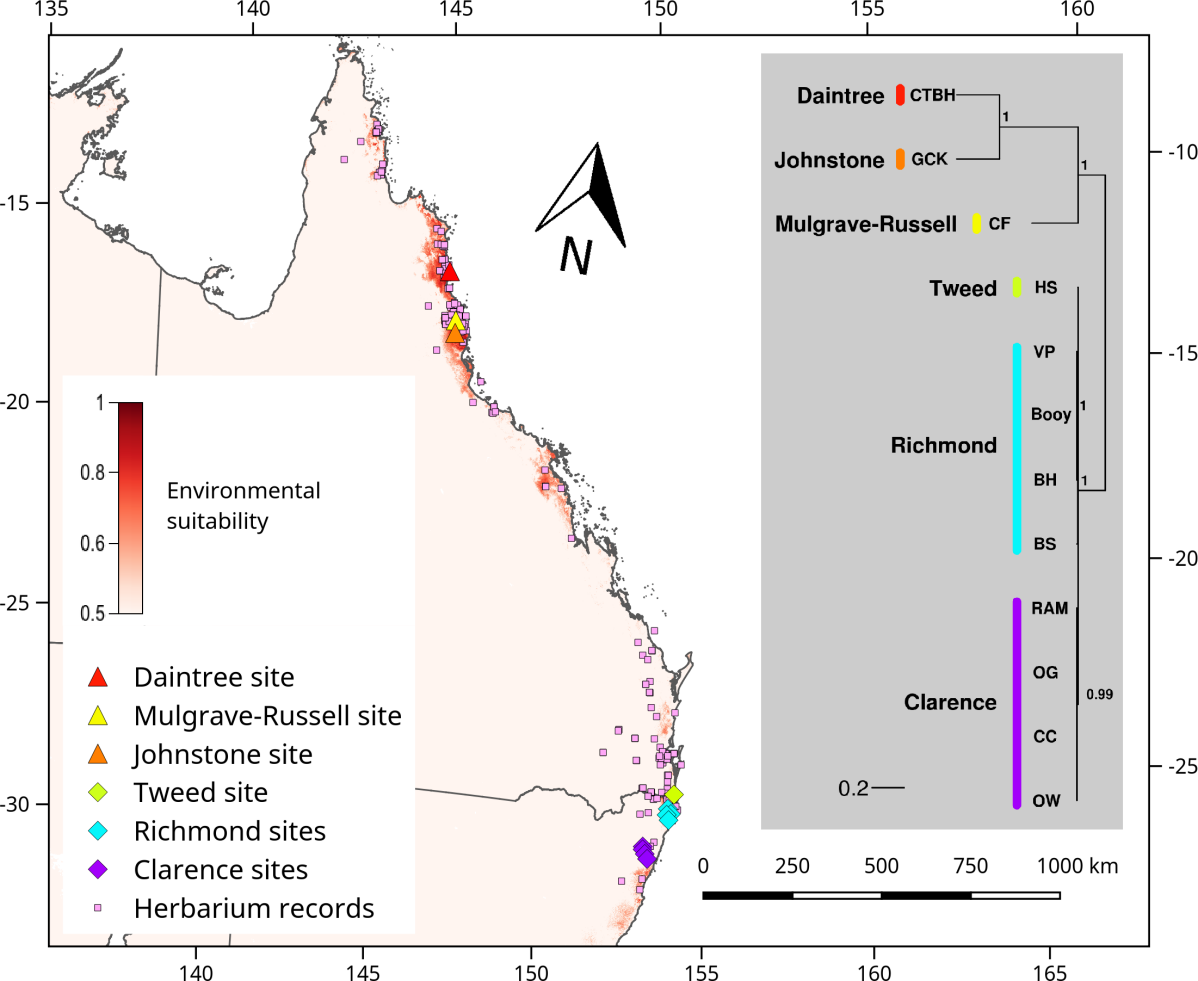
Geographical location, environmental niche model and distance-based relationship tree of sampled sites for *Castanospermum australe*. The map indicates the location of each sampled site within NNSW and AWT (each site represents eight individuals), with a different colour to represent each catchment, and pink squares to represent the herbarium records to display overall species’ distribution. Shading within the map represents a relative scale of environmental suitability for the study species. A distance tree shows the relative genetic distances between catchments (branch lengths according to indicated scale of nucleotide substitutions per base pair, node values indicate branch support), and identifies sites to each catchment (represented by the colour used in the map). The objective of the tree is not to estimate branching topology or deeper relationships among its branches, but to visually represent diversity within the focal lineage.

**Fig 2:**
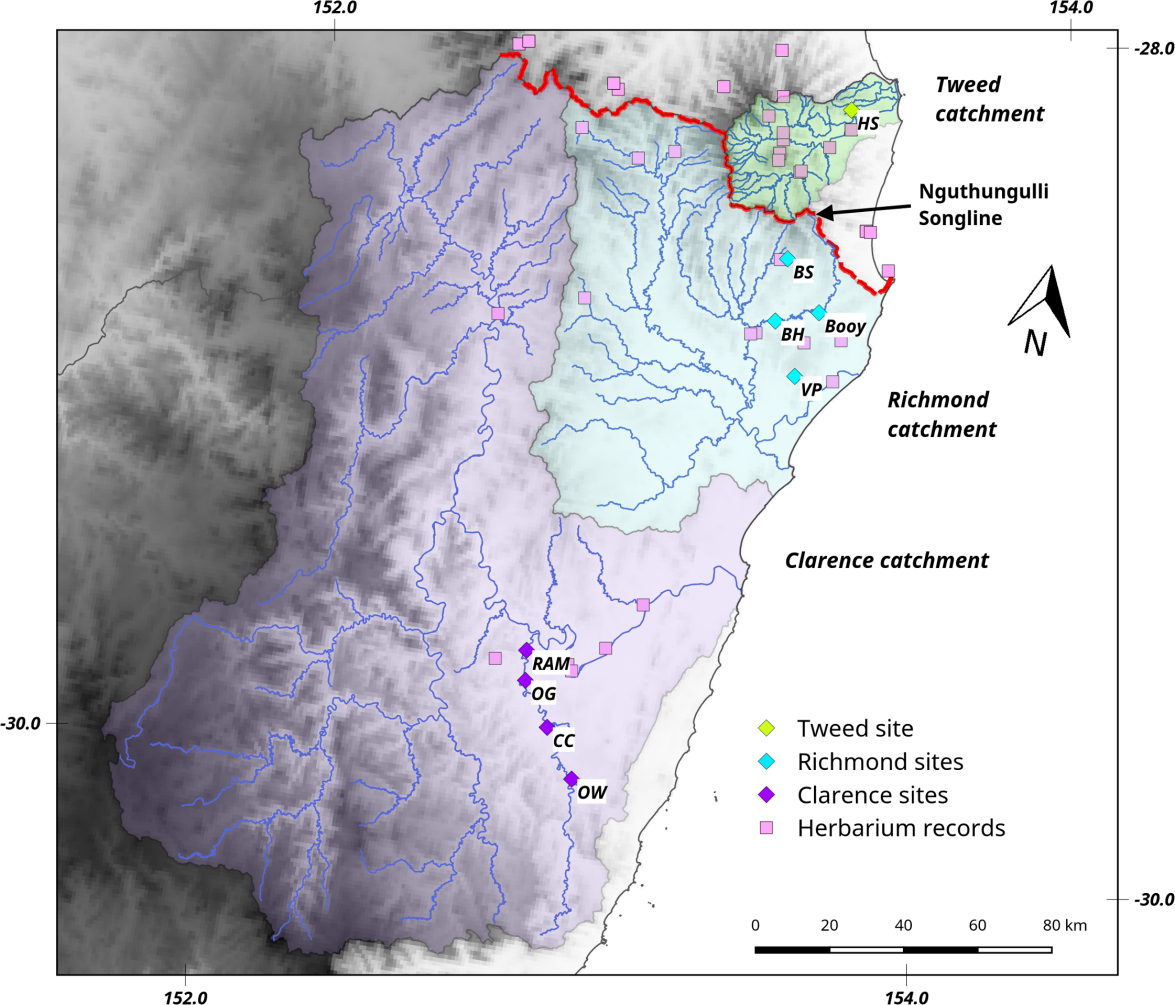
Map of northern NSW (NNSW) showing sampled sites, relevant river catchments, and Aboriginal Songline. The main study area (NNSW) showing: shaded elevation (darker equals higher); the distribution of *Castanospermum australe* (verified herbarium records, pink squares); the location of the genomic samples across the three main catchments (locations are colour-coded as per Fig 1); publicly listed Aboriginal places of importance; and the Nguthungulli Songline. The Songline traverses the main ridge of the Nightcap, Border and McPherson ranges, which traverse the top of the three main NNSW catchments across which *C*. *australe* was sampled for this study.

Our study integrates from the onset, anthropological, molecular and ecological research to test if Aboriginal-mediated dispersal can explain the distribution of a valuable but non-cultivated resource tree, *Castanospermum australe* A. Cunn. ex Mudie (black bean; Fabaceae). *Castanospermum australe* usually relies on hydrochory (water-dispersal) rather than ingestion-dependent mechanisms for range expansion [17]. It has large and buoyant seedpods, exposure to saltwater does not hinder the germination of its seed [17], and neither fruits nor the highly toxic seeds [18] enclose disperser rewards. The distribution of this tree along riparian habitats and close to the coast [19] reflects its reliance on water dispersal. However, multiple populations are located among rainforest vegetation that is inland from the coast and at considerable elevation (Fig 1 and Fig 2), implying dispersal through alternative means.

Traditionally, Australian Aboriginal people used the highly nutritious seeds of *C*. *australe* as a staple food source [20], [21] after extensive treatment to neutralise the toxins [20], [22]. Once ripe (May-July) the seeds were often stored underground for several months [23], [24]. Additionally, once prepared the ground *C*. *australe* “meal” could be stored for later use [22]. Although archaeological studies identified strong links between the application of seed detoxifying techniques and the more intensive occupation of rainforest habitats of northern Queensland between 2,500 and 1,000 years ago [25], [26], dispersal by Aboriginal people has not been previously investigated. We use an integrative exclusion-based framework to show that prehistoric Aboriginal-mediated dispersal explains the current distribution of *C*. *australe* within northern New South Wales (NNSW; Fig 2). We focused our attention on this geographically well-defined area because of the paucity of local disperser fauna (and absence of megafauna [27]), our understanding of local rainforest dynamic and biogeographic processes [28], [29], and because Aboriginal dispersal was proposed as a speculative explanation for the NNSW inland distribution of the species [22].

To determine if deliberate dispersal by local Aboriginal people influenced the contemporary distribution patterns of *C*. *australe*, we aim to: 1) reveal prehistoric, historic, linguistic and ethnographic cultural evidence of use and deliberate dispersal; 2) analyse chloroplast and nuclear (ribosomal) genomic data to detect the genetic signature of recent, rapid expansion within NNSW; 3) exclude alternative explanatory scenarios through the integration of complementary sources of evidence, and by comparing the NNSW findings to a representative sample from the Australian Wet Tropics (AWT), where ecological [27] and prehistoric cultural circumstances differ [25], [26].

## Materials & methods

### Study species and sampling strategy

*Castanospermum australe* (black bean) is distributed along coastal eastern Australia, from Cape York to subtropical northern New South Wales (Fig 1). It is a large riparian tree (to 40 m) well represented in the understorey of old-growth forest that can also be found in tree-fall gaps and disturbed habitats. Seedling physiology suggests that this species can act as an early-successional, as well as a mature-phase tree [30]. Black bean produces cauliflorous racemes 5–15 cm long, with orange to red flowers 30–40 mm long that attract both vertebrate and insect pollinators.

Seedpods, up to 20 cm long, contain three to five 3 cm wide seeds, and are buoyant and salt tolerant [17]. The seed comprises large cotyledons with a non-photosynthetic role. After germination, long-lived seedling banks can be established. The capacity of seeds to disperse across oceans is substantiated by the species’ distribution, which extends across a considerable area of the south-western Pacific, from Eastern Australia to New Caledonia and Vanuatu, and by close phylogenetic and phytochemical relationship between the monotypic *Castanospermum* and the South American rainforest genus *Alexa* (nine species [31], [32]). The seeds of *C*. *australe* contain high concentrations of alkaloids and saponins that can deter seed predation [33]. These compounds have received significant scientific attention in connection with their anti-viral properties [34], as well as in relation to their toxicity [18].

Our sampling strategy within the context of local Aboriginal dispersal focused on sites representing the species’ southern distributional margin in northern New South Wales (NNSW, Australia) and was not intended to exhaustively represent all populations to explore continent-wide connectivity, or broader biogeographic questions. Sampling was undertaken under a Scientific Licence (#100569), Section 132c of the National Parks and Wildlife Services Act 1974 issued by the New South Wales Office of Environment and Heritage (sampling did not involve endangered or protected species). The objective was to ensure sufficient geographic representation across the main NNSW catchment areas in order to investigate genomic homogeneity vs. genomic heterogeneity hypotheses. Highly homogeneous maternally-inherited plastid genomes across the study area, would suggest rapid and recent dispersal from a small founder event, while plastid heterogeneity would suggest lengthier local persistence and / or multiple founder events. The focus on NNSW was influenced by the current paucity of local disperser fauna and absence of megafauna [27], and by our understanding of local rainforest dynamics and the impact of environmental and biogeographic processes on species distribution and community assembly [28], [29].

Representative sites from the Australian Wet Tropics (AWT, northern Queensland, Australia) were also included as a comparative northern, latitudinally disjunct sample from the rainforest region with the highest flora and fauna diversity in Australia (including the remaining rainforest megafauna representative, the Cassowary: *Casuarius casuarius johsonii*). This northern area also has an extended archaeological record of the use of *C*. *australe*’s seeds [26].

Eight mature individuals were sampled from each site (for a total of 96 individuals across 12 sites), care was taken to avoid sites impacted by modern human activity and sampling was restricted to reserves or conservation areas (Table 1).

**Table 1:** *Castanospermum australe* sampling sites. Sites are arranged in a latitudinal pattern, starting with the comparative northern sites (AWT) followed by the main study sites (NNSW). Names (and abbreviations), location details, elevation and distance from the coast are listed.

Population ^a^	Catchment	Location ^b^	Elev. ^c^	Coast ^d^
**Australian Wet Tropics (AWT)**				
Cape Tribulation Beach House (CTBH; 8)	Daintree	-16.06933 145.46231	27 m	0.5 km
Curtain Fig (CF; 8)	Mulgrave-Russell	-17.28596 145.57435	605 m	25 km
Gooligan Creek (GCK; 8)	Johnstone	-17.60330 145.76876	378 m	35 km
**Northern New South Wales (NNSW)**				
Hogan’s Sands (HS; 8)	Tweed	-28.25315 153.44215	127 m	14 km
Big Scrub (BS; 8)	Richmond	-28.62808 153.33625	246 m	26 km
Booyong (Booy; 8)	Richmond	-28.74300 153.44711	25 m	17 km
Boat Harbour (BH; 8)	Richmond	-28.77973 153.33145	13 m	29 km
Victoria Park (VP; 8)	Richmond	-28.90231 153.41044	168 m	12 km
Ramornie (RAM; 8)	Clarence	-29.65213 152.79962	23 m	56 km
Old Glenn Innes Rd (OG; 8)	Clarence	-29.72352 152.81002	22 m	52 km
Coutts Crossing (CC; 8)	Clarence	-29.82578 152.89140	33 m	42 km
Orara Way (OW; 8)	Clarence	-29.93808 152.98257	54 m	26 km

^a^ Abbreviation and sample size for each population is listed in brackets.

^b^ Denotes approximate latitude and longitude of each site.

^c^ Altitude in metres extracted from high-resolution digital elevation data available from Geoscience Australia (www.ga.gov.au).

^d^ Distance from coast measured as the shortest straightline distance from the site to the coast using the measurement tool in QGIS (www.qgis.org).

### Anthropological data collection

A desktop literature search was conducted during 2015 and 2016 to compile historic documentation of early colonial European observations of Aboriginal use and movement of *C*. *australe* as well as any linguistic or cultural information (*e*.*g*., legends, Songlines). Sources searched included JSTOR, Google Scholar, Google, and the Australian Institute of Aboriginal and Torres Strait Islander studies MURA electronic databases using the scientific and common names (black bean, Moreton Bay chestnut, bean tree) and NNSW Aboriginal tribal names (Bundjalung, Githabul, Gidabul, Bandjalang, Widjabul and spelling variations of these names). We also investigated Aboriginal language dictionaries and any other historical documentation from NNSW including the private and extensive library of Tweed Valley Aboriginal history expert Ian Fox. References to significant Aboriginal sites, Songlines and pathways in NNSW were mapped using QGIS (version 2.18, www.qgis.org).

In 2016, five Aboriginal knowledge custodians from NNSW were interviewed about their knowledge of *C*. *australe*. Semi-structured interviews were facilitated by Oliver Costello and Emilie Ens and centred on the questions presented in S2 Appendix. Human Research Ethics approval was received from Macquarie University to record interviews about *C*. *australe*. All individuals in this manuscript and supporting information have given written informed consent (as outlined in PLOS consent form, and in a standard Macquarie University consent form) to publish these case details.

Although post-colonial non-Aboriginal terminology of Aboriginal knowledge, songs and dance are often described as “Dreaming” stories or legends, these forms of expression often contain knowledge that is founded on past observations, actions and lessons that have been encoded over millennia in orally transferrable and memorable forms. They should not be discounted as myths or fairy-tales. Western scientific thought has told us that the truth must be separated from religious or spiritual interpretations, which has resulted in modern misunderstanding and misinterpretation of many traditional or Indigenous knowledge systems across the world [35].

### Chloroplast genome and ribosomal DNA sequencing

Population dynamic processes mediated by different mechanisms produce distinguishable genetic patterns, particularly in relation to expansion across the landscape. The investigation of chloroplast DNA (cpDNA) variation across the landscape provides broadly applicable methods for quantifying seed-mediated dispersal across a range of spatial scales [36]. The maternal inheritance and conserved nature of chloroplast DNA make it particularly useful for exploring seed-mediated dispersal, although traditional sequencing approaches can provide limited analytical power [37]. Recently developed DNA-based approaches that enable the analysis of whole plastid genomes and other highly repetitive DNA sequences provide new opportunities for detecting landscape-level patterns, even in non-model species with low diversity [38], [39]. Here, we used genome skimming (defined as the low coverage shotgun sequencing of total DNA [40]) and bioinformatic analyses to capture Single Nucleotide Polymorphism (SNP) variation in chloroplast and nuclear ribosomal DNA [28].

Leaf tissue was sampled from 96 individuals (eight individuals from each of the 12 study sites according to the sampling strategy), and stored at -80°C prior to DNA extraction. Total DNA was extracted from each individual using Qiagen DNeasy Plant Mini kits and DNA samples were quantified using a Qubit 2.0 fluorometer (Life Sciences). To prepare site-specific genomic libraries, the eight DNA extracts obtained from each site were normalised and pooled for next generation sequencing. As our objective was to quantify nucleotide variation within and between sites rather than characterise individuals, we used pooled samples by site and measured within-site variation (*i*.*e*., SNPs detected within a site-specific pool) as well as variation among sites (*i*.*e*., fixed sequence differences between sites) [39]. Library preparation followed standard Nextera protocols (Illumina Inc., San Diego, CA, USA), and paired-end (2x150 bp) shotgun sequencing was performed on an Illumina Genome Analyser (GAIIx) by the Ramaciotti Centre for Genomics (University of NSW, Australia). Details of shotgun sequencing outputs are given in S5 Appendix.

Paired-end reads were imported into CLC Bio Genomics Workbench (version 6.5, www.clcbio.com) and sequences were trimmed using default settings to remove low quality reads and reads under 50 bp long. To enable read mapping and SNP detection, reference sequences representing the *C*. *australe* chloroplast genome and nuclear ribosomal DNA were assembled from each of the site-specific shotgun libraries following a standardised approach [39]. Reads from each library were mapped separately onto the constructed reference sequences with the mapping similarity and length fraction set to 0.8 and 0.9 respectively.

Within-population variant detection was conducted with minimum variant frequency set as the percentage contribution of each individual included in the pool (*i*.*e*., 12.5%). For variants to be confirmed a minimum coverage of 20x was used, and visual inspection verified their presence in both forward and reverse directions. Consensus sequences (representing each site) from the chloroplast and ribosomal read mappings were imported into Geneious Pro (R8, www.geneious.com, Biomatters Ltd.) for alignment. Areas of low coverage were removed and SNP location was annotated onto the consensus files [39].

In order to obtain a simple representation of between-population genomic distances, pairwise distances were computed using the MAFFT plugin in Geneious with default settings. A chloroplast sequence alignment of all *C*. *australe* sites was analysed using the MrBayes plugin in Geneious [41], and a relationship tree was generated using gamma-distributed rate variation and an HKY85 substitution model, with the first 100,000 of a 5,000,000 chain-length discarded as burn-in, and four heated chains were run with a subsampling frequency of 5,000. The objective of the tree was not to estimate branching topology or deeper relationships among its branches, but to visually represent diversity within the focal lineage (Fig 1).

As well as within-population diversity and between-population distance measures, we estimated within-catchment diversity and between-catchment distance. Within-catchment SNP diversity was determined by the total number of fixed SNPs found only within a catchment but not in other catchments (*i*.*e*., number of fixed, unique SNPs). Average number of between-catchment SNPs (as well as average number of SNPs differentiating between NNSW and AWT) was determined by the total number of fixed SNPs that differentiated catchments (or regions) from one another [28].

## Results & discussion

### Anthropological evidence of use and dispersal of black bean seeds

We reveal anthropological evidence for prehistoric Aboriginal-mediated dispersal by verifying that: Aboriginal people used the species; and several sources including Songlines (Dreaming tracks) describe the deliberate movement of this species by Aboriginal people. Linguistic evidence could neither support nor negate the hypothesis of human dispersal and lateral language transfer.

We found numerous historical records from the early Australian colonial period [22], [26] describing detoxification and food preparation methods of *C*. *australe* seed by NNSW Aboriginal people. Numerous ethnographic records also describe other uses of *C*. *australe* by Aboriginal people from the AWT [20], [21], [23] including: using bark fibre for fish and animal traps, nets and baskets; wood for spear throwers; seed pods as toy boats; and as a seasonal cue for jungle fowl hunting. We corroborated the usefulness of black bean as a food resource through ethnographic interviews with five NNSW Aboriginal knowledge custodians in 2016 (S1 and S2 Appendices).

Recent mitochondrial DNA studies revealed that Aboriginal people have inhabited Australia in consistent geographic arrangements for up to 50,000 years [42]. Continuous regional persistence facilitated the strong connection to country by local Aboriginal communities who, through time, maintained knowledge via oral transmission. Traditional Aboriginal Songlines (Dreaming stories / tracks) are physical pathways that were traversed by Aboriginal people and for which specific songs and stories were told to pass on and maintain knowledge.

We recovered three Dreaming stories of the movement, maintenance and significance of *C*. *australe* in NNSW (S1 Appendix), of which the Nguthungulli Songline told by Ngarakbal woman Charlotte Brown and recorded by Roland Robinson in the 1950’s [43] is the most pertinent. This story tells that Nguthungulli (an ancestral spirit likely to represent a real person) carried and left “bean tree” (*C*. *australe* is the only local species being commonly referred to as ‘bean tree’) seeds as he journeyed inland from the east coast to the western Ranges (S1 Appendix point 1.2). In our study, the Songline was traced for the first time on a topographic map by local Aboriginal man, Oliver Costello, and traditional pathway expert, Ian Fox (Fig 2). Based on their intimate cultural and migration knowledge of the region they believe that like many Aboriginal pathways which followed points of high elevation for ease of access and vision, the Nguthungulli Songline traverses the ridges of the Nightcap Border and McPherson ranges dividing New South Wales from Queensland (Fig 2). The relevance of this pathway is that the northern ridges of the Nightcap ranges correspond to the top of the drainage basin for the Tweed River, while the southern faces of the Border and McPherson ranges correspond to the top of the drainage basins for the Richmond and Clarence Rivers. These catchments represent our sampling sites for the genomic analyses (Fig 2). From an Aboriginal knowledge perspective, these stories confirm that in prehistoric times Aboriginal people used black bean seeds as food, and deliberately moved them around the landscape including along the ridgelines of NNSW.

Although previous studies have used language to support human-dispersal scenarios [15], linguistic data can neither confirm nor negate human dispersal of *C*. *australe* within NNSW. Histories of individual words are shaped by vertical inheritance, language-internal replacements, slowly accumulated sound mutations, shifts in meaning, or lateral transfer. The geographic range of *C*. *australe* coincides with the Indigenous languages of the Pama-Nyungan language family [44]. We identified linguistic terms for *C*. *australe* in eight linguistic subgroups of the eastern clade of the Pama-Nyungan language family (S3 Appendix).

The languages of NNSW are included within the Bandjalangic clade, whose most recent common ancestor (MRCA) is likely to be close to 1,500 years old (C. Bowern *pers*. *comm*.). Common inheritance from the MRCA results in Bandjalangic vocabulary being inherited with only few sound changes [45], and the word for *C*. *australe* is uniformly *bugam*. This is in contrast with the rest of the distribution of *C*. *australe* where the terms exhibit little homology, even within members of the same low-level clade such as the Djirbalic languages from the AWT (S3 Appendix).

The absence of mutation of any of the phonemes in *bugam* prevents us from positively diagnosing inheritance with modification, but is also in line with a scenario of locally recent dispersal of black bean. The divergence with words for *C*. *australe* from both neighbouring and more distant language clades suggest that lateral transfer of *bugam* into post-MRCA Bandjalangic from external sources is improbable. Thus, while alternatives are possible, it is most likely that as the Bandjalangic languages expanded into their current territory or diversified in situ, they inherited *bugam* continuously from their MRCA.

### Genomic evidence of rapid, recent and widespread expansion in NNSW

The combination of ethnographic data and first-hand corroboration by Aboriginal knowledge custodians from NNSW confirmed that *C*. *australe* was a valuable local traditional resource plant and was likely to have been intentionally moved by Aboriginal people across the focus area of this study. We further interpreted the NNSW distribution of this tree within the framework of aboriginal-mediated dispersal by sampling local genomic variation.

We used genomic analyses to search for a signature of the rapid local expansion expected from the human-mediated timeline set by archaeological evidence on the use of seed detoxification techniques [25]. Single nucleotide polymorphism (SNP) detection from low-coverage genome sequencing can reveal small amounts of within- and between-population variation that enables the exploration of fine-scale dispersal patterns even in non-model species with low diversity [38], [27].

A total of 124,678bp of chloroplast DNA (cpDNA) and 5,813bp of nuclear ribosomal DNA (nrDNA) were analysed and compared among *C*. *australe* sites (comprising eight individuals each), catchments and regions. Analyses of sequence data across 96 individual trees from 12 distinct sites yielded 987 cpDNA SNPs (0.79% of sequence analysed) and 29 nrDNA SNPs (0.50% of sequence analysed). None of these 12 sites yielded within-site cpDNA variability, suggesting that all eight individuals sampled within each population originated from a single maternal lineage. The nrDNA sequences produced between four and 12 heterozygotic variants across 5,813bp, confirming a pattern of low within-population diversity (Table 2).

**Table 2:** Summary of cpDNA and nrDNA genomic data for *Castanospermum australe*. Population-level diversity for chloroplast and nuclear ribosomal DNA across all sample sites, and regional between-catchment genomic distances. Overall, diversity and divergence are higher in the comparative northern populations (AWT), than across the main study sites in northern NSW.

Within-catchment diversity	N^a^	cpDNA SNPs ^b^ (SNP per bp) ^c^	nrDNA SNPs ^b^ (SNP per bp) ^c^	nrDNA average within-population variants ^d^
**AWT catchment sites**				
Daintree	1 (8)	166 (1.3x10-3)	2 (3.0x10-4)	7
Mulgrave-Russel	1 (8)	161 (1.3x10-3)	0 (0)	7
Johnstone	1 (8)	182 (1.5x10-3)	1 (1.7x10-4)	8
**NNSW catchment sites**	** **	** **	** **	** **
Tweed	1 (8)	0 (0)	0 (0)	4
Richmond	4 (32)	4 (3.2x10-5)	0 (0)	12
Clarence	4 (32)	2 (1.6x10-5)	0 (0)	9.5
**Between-catchment genomic distances**	**N**^a^	**cpDNA** ^e^ **(N SNPs)** ^f^	**nrDNA** ^e^ **(N SNPs)** ^f^	** **
Average within AWT	3 (24)	4.1x10^-3^ (507)	1.7x10^-4^ (1)	
Average within NNSW	9 (72)	1.0x10^-4^ (13)	0 (0)	
Average AWT vs. NNSW	12 (96)	4.1x10^-3^ (511)	3.4x10^-4^ (2)	

^a^ Number of site/s and, in brackets, number of individuals sampled.

^b^ Number of within-catchment SNPs that are fixed and not found in other catchments (*i*.*e*. unique to the catchment).

^c^ SNP per base pair (based on total assembled cpDNA sequence 124,678 bp; and total assembled nrDNA sequence 5,813 bp).

^d^ Average within-population number of heterozygous nrDNA sites for each catchment. No within-population variants were found in cpDNA.

^e^ Average pairwise genomic distance between catchments.

^f^ Average number of fixed between-catchment SNPs (*i*.*e*., differentiating catchments).

In water-dispersed species, landscape-level constraints can result in the localised patterns of relatedness and population-level cpDNA uniformity observed across the distribution of *C*. *australe* [46]. However, in NNSW genomic homogeneity extended across whole catchments, with every site sampled from within each catchment belonging to the same lineage (regardless of geographic distances and topography; Fig 2, Table 2). The only exception was one unique, fixed SNP found in the Big Scrub site within the Richmond catchment. For hydrochory to generate homogeneous cpDNA patterns within catchments, landscape conditions need to favour long-distance and directed seed dispersal along water-bodies [47]. However, the altitudinal heterogeneity and geographic scale that typifies the southern distribution of *C*. *australe* is unlikely to favor rapid water-mediated inland expansion (Fig 2).

Hydrochory can also reduce spatial aggregation of genetically related individuals resulting in high genetic divergence between catchments [46], [47]. While the comparative samples from the northern (AWT) distribution of *C*. *australe* fit the expected pattern of high between-catchment differentiation, the NNSW sites do not. High diversity was detected in the north (Fig 1, Table 2), with considerably more unique SNPs present across three AWT sites (509 in cpDNA, and 3 in nrDNA) than across nine NNSW sites (6 in cpDNA, and none in nrDNA). Genomic homogeneity in NNSW extended across three catchments covering a large (30,604 km^2^) and topographically complex area (from sea level to 1,166 m across multiple dissected ranges; Fig 2, Tables 1 and 2) crossing the Clarence River Corridor (into the Orara catchment; Fig 2), an important biogeographic barrier that can restrict genetic connectivity even in easily-dispersed species [28].

This implies that, unlike in the comparative sample from the AWT (high diversity among three adjacent catchments covering a smaller area of 6,550 km^2^; altitude sea level to 783 m; Table 1), all sampled NNSW populations are derived from recent dispersal events sourced from one or a small number of very closely related maternal lineages (Fig 1). The non-accrual of unique, catchment-specific nrDNA variation (Table 2) further supports rapid expansion within the southern range of the species, as the accumulation of detectable distinctive mutations is expected from ancient processes [48]. These findings match the population genetic expectations from the recent Aboriginal dispersal scenario suggested by anthropological, cultural and linguistic evidence.

### Excluding alternative rapid, southern expansion scenarios

Although other interpretations could be proposed for some aspects of the data presented, when combined our findings identify recent Aboriginal dispersal as the most parsimonious explanation for the current NNSW distribution of *C*. *australe*. Landscape genetics patterns suggesting recent, rapid population expansions have also been observed in natural post-glacial settings [49]. In a natural post-glacial expansion scenario, *C*. *australe* would have been restricted to a small, refugial population within NNSW during the Last Glacial Maximum (LGM), and a single founder lineage would have rapidly expanded to its current distribution as habitat became increasingly available. Environmental niche models representing the availability of climatic conditions suitable to *C*. *australe* during the LGM suggest that available habitat is likely to have increased in the current interglacial period, and that environmental suitability in the south remains marginal compared to the north (Fig 2, S4 Appendix). The modelled increase in suitable habitat fits both the recent human-mediated dispersal proposition (following the pursuit of newly available resources [25]), and the natural expansion scenario (as proposed for other local trees [38], [50]). However, the latter processes invariably rely on efficient dispersal mechanisms that are not available to *C*. *australe* in NNSW.

Along coastal areas and low-lying waterways, the contribution of rapid, recent water-mediated geographic expansion (potentially even derived from a northern source) cannot be excluded. However oceanic currents, tidal processes, extreme weather events or river capture cannot explain the location of multiple sites inland and well above current and historical sea level [51]. Secondary movement of water-dispersed species inland away from the riparian zone, to upland areas, or between catchments is commonly performed by animals [47].

Within Australian rainforests, the limited number of functional classes in fruit-dispersing fauna can restrict the movement of large-seeded species irrespectively of habitat availability [27]. Consequently, the distribution and assembly of Australian rainforest plants is impacted by fruit type and size. Species producing small, palatable fleshy fruits occupy significantly larger geographical ranges than species producing poorly dispersible fruits, and recolonised landscapes lack the large-fruited component of the flora [27]. An animal dispersal (zoochory) hypothesis for the current distribution of *C*. *australe* is therefore at odds with its large, toxic and reward-free seeds. Even within the AWT, where the highest diversity of frugivorous animals persists (including the remaining rainforest megafauna representative, the Cassowary), the high genetic divergence measured between neighbouring catchments (Fig 1, Table 2) suggests that zoochory does not play a critical role in maintaining connectivity among *C*. *australe* populations. An alternative scenario involving the potential contribution of now-extinct megafauna is also doubtful, as the timeframes involved in the loss of large rainforest vertebrates are too extended [52] to justify the genomic homogeneity detected in NNSW.

## Conclusions

The new, combined evidence presented supports the deliberate dispersal of *C*. *australe* by prehistoric Aboriginal people as the most parsimonious interpretation for the species’ distribution in NNSW. Ferrier & Cosgrove [26] suggested that in the AWT, the expansion of Australia’s rainforest-dwelling people during the late Holocene (between 2,500 and 1,000 years ago) could have been facilitated by the development of detoxifying techniques for rainforest nuts such as *C*. *australe*, which became critical technologies aiding survival in areas where these resources were available. We demonstrated that the inverse is also true: local Aboriginal communities developed food preparation technologies that had a deliberate impact on the distribution of culturally important rainforest species. In Australia, with the exception of obvious fire-related impacts, Aboriginal influence on the distribution and assembly of species was largely ignored by early European colonists. Our and similar studies can help expand cultural heritage management practices by promoting the need to maintain living biotic heritage (in this case *C*. *australe* groves) in collaboration with Aboriginal knowledge custodians.

Evidence of prehistoric Australian Aboriginal people dispersing plant propagules for their direct need and benefit also significantly challenges assumptions of “natural” plant distributions, requiring reassessment of distributional interpretations that omit the possible impact of prehistoric human intervention [6]. Current debates on the role of human-assisted migration [53] and other active management options could also benefit from the acceptance, from conservation practitioners and the general public, that Aboriginal people deliberately dispersed species in the past. This is particularly relevant since current measures of restoration success are often based on historical (pre-European) reference systems.

Now that the role of Aboriginal dispersal has been established regionally for *C*. *australe*, future studies can explore broader biogeographic questions including possible dispersal routes along eastern Australia and across the Pacific Islands. A scenario of long-distance dispersal by ancestors from the north was independently put forward by Aboriginal knowledge custodian Uncle Ron Heron (S1 Appendix, point 1.7).

## Supporting information

S1 Appendix Selected cultural data examples. (DOCX)

S2 Appendix Interview questions and anthropological evidence. (DOCX)

S3 Appendix Linguistic data. (DOCX)

S4 Appendix Environmental niche models. (DOCX)

S5 Appendix Additional genomic information. (DOCX)

S6 Appendix Short video showing a brief summary of a workshop held in early 2017, and aimed at bringing together Indigenous communities from the east coast of Australia to share and revive knowledge on Black Bean processing as a staple food item. (MP4)

## References

[1] Bowman DMJS. The impact of Aboriginal landscape burning on the Australian biota. New Phytol. 1998; 140: 385–410. 10.1111/j.1469-8137.1998.00289.x 33862877

[2] Ens E, Walsh F, Clarke P. Aboriginal people and Australia’s vegetation: past and current interactions In: Keith D, editor. Australian vegetation. Cambridge: Cambridge University Press; 2017.

[3] Miller GH, Fogel ML, Magee JW, Gagan MK, Clarke SJ, Johnson BJ. Ecosystem collapse in Pleistocene Australia and a human role in megafaunal extinction. Science. 2005; 309: 287–290. doi: 10.1126/science.1111288 16002615 10.1126/science.1111288

[4] Shepard GH, Ramirez H. “Made in Brazil”: Human dispersal of the Brazil nut (*Bertholletia excelsa*, Lecythidaceae) in ancient Amazonia. Econ. Bot. 2011; 65: 44–65.

[5] Thomas E, Alcázar Caicedo C, McMichael CH, Corvera R, Loo J. Uncovering spatial patterns in the natural and human history of Brazil nut (Bertholletia excelsa) across the Amazon Basin. J. of Biog. 2015; 42: 1367–1382.

[6] Levis C, Costa F, Bongers F, Peña-Claros M, Clement CR, Junqueira AB, et al Persistent effects of pre-Columbian plant domestication on Amazonian forest composition. Science. 2017; 355: 925–931. doi: 10.1126/science.aal0157 28254935 10.1126/science.aal0157

[7] Zerega NJC, Ragone D, Motley TJ. Complex origins of breadfruit (*Artocarpus altilis*, Moraceae): implications for human migrations in Oceania. Am. J. Bot. 2004; 91: 760–766. doi: 10.3732/ajb.91.5.760 21653430 10.3732/ajb.91.5.760

[8] Clarke AC, Burtenshaw MK, McLenachan PA, Erickson DL, Penny D. Reconstructing the origins and dispersal of the Polynesian bottle gourd (*Lagenaria siceraria*). Mol. Biol. and Evol. 2006; 23: 893–900. 16401685 10.1093/molbev/msj092

[9] Roullier C, Benoit L, McKey DB, Lebot V. Historical collections reveal patterns of diffusion of sweet potato in Oceania obscured by modern plant movements and recombination. Proc. Natl Acad. Sci. 2013; 110, 2205–2210. doi: 10.1073/pnas.1211049110 23341603 10.1073/pnas.1211049110 PMC3568323

[10] Perrier X, Langhe ED, Donohue M, Lentfer C, Vrydaghs L, Bakry F, et al Multidisciplinary perspectives on banana (*Musa* spp.) domestication. Proc. Natl Acad. Sci. 2011; 108: 11311–11318. doi: 10.1073/pnas.1102001108 21730145 10.1073/pnas.1102001108 PMC3136277

[11] Hynes RA, Chase AK. Plants, sites and domiculture: Aboriginal influence upon plant communities in Cape York Peninsula. Archaeol. Ocean. 1982; 17: 38–50.

[12] Atchison J. Human impacts on *Persoonia falcata*. Perspectives on post-contact vegetation change in the Keep River region, Australia, from contemporary vegetation surveys. Veg. Hist. Archaeobot. 2009; 18: 147–157.

[13] Kondo T, Crisp MD, Linde C, Bowman DMJS, Kawamura K, Kaneko S, et al Not an ancient relic: the endemic *Livistona* palms of arid central Australia could have been introduced by humans. Proc. Biol. Sc. 2012; 279: 2652–2661. 22398168 10.1098/rspb.2012.0103 PMC3350701

[14] Bell KL, Rangan H, Fowler R, Kull CA, Pettigrew JD, Vickers CE, et al Genetic diversity and biogeography of the boab *Adansonia gregorii* (Malvaceae: Bombacoideae). Aust. J. Bot. 2014; 62: 164–174.

[15] Rangan H, Bell KL, Baum DA, Fowler R, McConvell P, Saunders T, et al New genetic and linguistic analyses show ancient human influence on Baobab evolution and distribution in Australia. PloS one. 2015; 10: e0119758 doi: 10.1371/journal.pone.0119758 25830225 10.1371/journal.pone.0119758 PMC4382155

[16] Bowman DMJS, Gibson J, Kondo T. Outback palms: Aboriginal myth meets DNA analysis. Nature. 2015; 520: 33–33. 10.1038/520033a 25832394

[17] Smith JMB, Heatwole H, Jones M, Waterhouse BM. Drift disseminules on cays of the Swain Reefs, Great Barrier Reef, Australia. J. Biogeogr. 1990; 17: 5–17.

[18] McKenzie RA, Reichmann KG, Dimmock CK, Dunster PJ, Twist JO. The toxicity of *Castanospermum australe* seeds for cattle. Aust. Vet. J. 1988; 65: 165–167. 3261977 10.1111/j.1751-0813.1988.tb14291.x

[19] Swanborough PW, Doley D, Keenan RJ, Yates DJ. Photosynthetic characteristics of *Flindersia brayleyana* and *Castanospermum australe* from tropical lowland and upland sites. Tree Physiol. 1998; 18: 341–347. 12651374 10.1093/treephys/18.5.341

[20] Birtles TG. In: Dargavel J, editor. Australia's ever-changing forests III: Proceedings of the third national conference on Australian forest history, Centre for Resource and Environmental Studies. Canberra: The Australian National University Press; 1997. pp. 169–187.

[21] Dixon RMW. Searching for aboriginal languages: Memoirs of a field worker Cambridge: Cambridge University Press; 1983.

[22] Maiden JH. Native food-plants. Agricultural Gazette of New South Wales. 1899; 10: 117–130, 279–190, 618–139, 730–140.

[23] Coyyan (M. O’Leary) 1918. The Aboriginals. Columns I-X. The Northern Herald (The Tablelander).

[24] Mjöberg, E. 1918. Bland stenåldersmänniskor i Queenslands vildmarker (Amongst Stone Age People in the Queensland Wilderness). Albert Bonniers Boktryckeri, Stockholm.

[25] Cosgrove R, Field J, Ferrier Ǻ. The archaeology of Australia’s tropical rainforests. Palaeogeogr. Palaeoclimatol. Palaeoecol. 2007; 251: 150–73.

[26] Ferrier Å., Cosgrove R. Aboriginal exploitation of toxic nuts as a late Holocene subsistence strategy in Australia’s tropical rainforests In: Haberle S G, David B, editors. Peopled landscapes. Archaeological and biogeographic approaches to landscapes. Canberra: The Australian National University Press; 2012 pp. 103–120.

[27] Rossetto M, Yap JYS, Kooyman R, Laffan S. From ratites to rats: the size of fleshy fruits shapes species distributions and continental rainforest assembly. Proc. R. Soc. B. 2015; 282: 20151998 doi: 10.1098/rspb.2015.1998 26645199 10.1098/rspb.2015.1998 PMC4685777

[28] Rossetto M, McPherson H, Siow J, Kooyman R, van der Merwe M, Wilson PD. Where did all the trees come from? A novel multidisciplinary approach reveals the impacts of biogeographic history and functional diversity on rain forest assembly. J. Biogeogr. 2015; 42: 2172–2186.

[29] Kooyman R, Rossetto M, Cornwell W, Westoby M. Phylogenetic tests of community assembly across regional to continental scales in tropical and sub-tropical rainforests. Glob. Ecol. Biogeogr. 2011; 20: 707–716.

[30] Osunkoya OO, Ash JE, Hopkins ME, Graham AW. Influence of seed size and seedling ecological attributes on shade-tolerance of rain-forest tree species in Northern Queensland. J. Ecol. 1994; 82: 149–163.

[31] Nash RJ, Fellows LE, Dring JV, Stirton CH, Carter D, Hegarty MP, Bell EA. Castanospermine in *Alexa* species. Phytochemistry. 1988; 27: 1403–1404.

[32] Cardoso D, Pennington RT, De Queiroz LP, Boatwright JS, Van Wyk BE, Wojciechowski MF, Lavin M. Reconstructing the deep-branching relationships of the papilionoid legumes. S. Afr. J. Bot. 2013; 89: 58–75.

[33] Osunkoya OO. Postdispersal survivorship of North Queensland rainforest seeds and fruits: effects of forest, habitat and species. Aust. J. Ecol. 1994; 19: 52–64.

[34] Whitby K, Pierson TC, Geiss B, Lane K, Engle M, Zhou Y, et al Castanospermine, a potent inhibitor of dengue virus infection *in vitro* and *in vivo*. J. Virol. 2005; 79: 8698–8706. doi: 10.1128/JVI.79.14.8698-8706.2005 15994763 10.1128/JVI.79.14.8698-8706.2005 PMC1168722

[35] Wehi PM, Whaanga H, Roa T. Missing in translation: Maori language and oral tradition in scientific analyses of traditional ecological knowledge (TEK). J. R. Soc. N. Z. 2009; 39: 201–204.

[36] Cain ML, Milligan BG, Strand AE. Long distance seed dispersal in plant populations. Am. J. Bot. 2000; 87: 2117–2227. 10991892

[37] Schaal BA, Hayworth DA, Olsen KM, Rauscher JT, Smith WA. Phylogeographic studies in plants: problems and prospects. Mol. Ecol. 1998; 7: 465–474.

[38] McPherson H, van der Merwe M, Delaney SK, Edwards MA, Henry RJ, McIntosh E, et al Capturing chloroplast variation for molecular ecology studies: a simple next generation sequencing approach applied to a rainforest tree. BMC Ecol. 2013; 13: 1–11. doi: 10.1186/1472-6785-13-1 23497206 10.1186/1472-6785-13-8 PMC3605380

[39] van der Merwe M, McPherson H, Siow J, Rossetto M. Next Gen phylogeography of rainforest trees: exploring landscape-level cpDNA variation from whole-genome sequencing. Mol. Ecol. Resour. 2014; 14: 199–208. doi: 10.1111/1755-0998.12176 24119022 10.1111/1755-0998.12176

[40] Coissac E, Hollingsworth PM, Lavergne S, Taberlet P. From barcodes to genomes: extending the concept of DNA barcoding. Mol. Ecol. 2016; 25: 1423–1428. doi: 10.1111/mec.13549 26821259 10.1111/mec.13549

[41] Huelsenbeck JP, Ronquist F. MRBAYES: Bayesian inference of 589 phylogenetic trees. Bioinformatics. 2001; 17: 754–755. 11524383 10.1093/bioinformatics/17.8.754

[42] Tobler R, Rohrlach A, Soubrier J, Bover P, Llamas B, Tuke J, et al Aboriginal mitogenomes reveal 50,000 years of regionalism in Australia. Nature. 2017; doi: 10.1038/nature21416 28273067 10.1038/nature21416

[43] Robinson RE. The Nearest the White Man Gets: Aboriginal narratives and poems of New South Wales. Indiana University: Hale & Iremonger; 1989.

[44] Bowern C, Atkinson Q. Computational phylogenetics and the internal structure of Pama-Nyungan. Language. 2012; 88: 817–845.

[45] Crowley T, Smythe WE. The Middle Clarence Dialects of Bandjalang. Canberra: The Australian Institute of Aboriginal Studies; 1978.

[46] Kondo T, Nakagoshi N, Isagi Y. Shaping of genetic structure along Pleistocene and modern river systems in the hydrochorous riparian azalea, *Rhododendron ripense* (Ericaceae). Am. J. Bot. 2009; 96: 1532–1543. doi: 10.3732/ajb.0800273 21628298 10.3732/ajb.0800273

[47] Nilsson C, Brown RL, Jansson R, Merritt DM. The role of hydrochory in structuring riparian and wetland vegetation. Biol. Rev. 2010; 85: 837–858. doi: 10.1111/j.1469-185X.2010.00129.x 20233190 10.1111/j.1469-185X.2010.00129.x

[48] Ossowski S, Schneeberger K, Lucas-Lledó JI, Warthmann N, Clark RM, Shaw RG, et al The rate and molecular spectrum of spontaneous mutations in *Arabidopsis thaliana*. Science. 2010; 327: 92–94. doi: 10.1126/science.1180677 20044577 10.1126/science.1180677 PMC3878865

[49] Hewitt G. The genetic legacy of the Quaternary ice ages. Nature. 2000; 405: 907–913. doi: 10.1038/35016000 10879524 10.1038/35016000

[50] Rossetto M, Jones R, Hunter J. Genetic effects of rainforest fragmentation in an early successional tree (*Elaeocarpus grandis*). Heredity. 2004; 93: 610–618. doi: 10.1038/sj.hdy.6800585 15367910 10.1038/sj.hdy.6800585

[51] Helman P, Thomalia F, Metusela C. Tomlinson, Storm tides, coastal erosion and inundation Cold Coast, Queensland: National Climate Change Adaptation Research Facility; 2010.

[52] Johnson C. Australia's mammal extinctions: a 50,000-year history Cambridge: Cambridge University Press; 2006.

[53] Corlett RT. Restoration, reintroduction and rewilding in a changing world. Trends Ecol. Evol. 2016; 31: 453–462. doi: 10.1016/j.tree.2016.02.017 26987771 10.1016/j.tree.2016.02.017

