# Supplementary material for: From Songlines to genomes: Prehistoric assisted migration of a rain forest tree by Australian Aboriginal people
Source: PLoS One. 2017 Nov 8;12(11):e0186663. doi: 10.1371/journal.pone.0186663 (PMC5695580; doi:10.1371/journal.pone.0186663)
Supplement: S1 Appendix — (DOCX) [file pone.0186663.s001.docx]

**S1 Appendix: Selected cultural data examples**

Relevant cultural information obtained during this study indicating: **1.1** detoxification of the seed for human consumption; **1.2**. & **1**.**3**. deliberate movement of *C. australe*; **1**.**4**. a sacred grove of *C. australe*; **1**.**5**. use of *C. australe* seed pods as toy boats; **1**.**6**. survival resources provided by *C. australe*; and **1**.**7**. dispersal of black bean from Northern Queensland to NNSW.

**1.1** Process of detoxification of the Black Beans seeds told by Uncle Roy Heron (recorded by Emilie Ens or EE for short).

“Black beans, you put ‘em on a rock and you give them a few stabs with a stone to mash them up a little bit. Not to smithereens you know, just a little but to break through that little cap. Then you put them in a smaller dilly [string] bag…and put the dilly bag in the running stream…for about 4-5 days. You know and that leaches out then...all of the poisonous and makes that food in there wholesome so all the poison is out. Now when it goes 5 or 6 days or 4 to 5 days the dilly bag is emptied on a river bank or on a stream bank on the stones and they sort of drip dry you know. So when they drip dry then they are bashed up again and put in a little rock holder bashed up again like you do with mashed potatoes, you know how you mash ‘em up? Then they are easy then to make into, with your hands into what would you call it, like a rissole, and then you take that rissole of the black bean and put in in the hot ash and they cook that way for so many minutes…all depends how big the fire is. But in the ash part and then they turn it over and left it there for so many minutes then they are done then, and that acts as a bread.”

**1.2**. Nguthungulli Songline [43]

“*A big rock stands in the sea six miles from Byron Bay. A man named Nguthungulli made that rock. Nguthungulli is our Father. He’s the Father of the whole world. Nguthungulli had a cave in the rock. After he made the rock, he told the four fairy woman, ‘gilarmavell’, to stay there. Then he went away from there and walked inland from the coast. He travelled over the mountains with his dog Korung. He had a walking stick, and when he put the stick into the ground He left behind a stone like a basin [Wollumbin, Mt Warning]. As he travelled He left the bean-trees [*C. australe*], and He named the different places on His journey. He named Woodenbong [where we are now], Nguthumbung. He went over the range to the head of the Condamine. When he knelt down at the river to drink, He left his handprints there in the stone*”.

**1.3**. Movement of *C. australe* in the opposite direction from west to east in NNSW (<http://www.visitrichmondvalley.com.au/pages/indigenous-culture>, likely told by Billy Walker).

“*The people of the Bundjalung tribe lived in this area for 6,000 years before the coming of the white man. Many who lived inland would make a journey to the coast during winter months when mullet was plentiful. They would bring with them seeds from the black bean tree [*C. australe*] for food and for trading. Some of these would be dropped as they made their way along the banks of the Richmond River to the coast*.”

**1.4**. A documented Aboriginal legend from the Kyogle district (inland NNSW) told by M. T. Close to Mildred Norledge, tells of a sacred patch of *C. australe* that is more sacred than others [54]. This could be an initial source population of this species that was required to be protected to maintain stock of the high volume food resource.

“*Within the tribal grounds of the Kyogle tribes there are growing along the banks of the river many bean trees, and there is one place where the bean trees flourish and grow where the trees are more sacred to the Witches than the bean trees growing elsewhere. Sacred are all bean trees to the Witches, and none may touch them, least of all in the place where the trees that are more sacred grow, for if people eat the bean of the tree the witches will punish that do so with DEATH*.” [54]

**1.5**. Constance Petrie [55] recorded a Dreaming story told to her by her father (a non-Aboriginal man who grew up in South East Queensland and NNSW in the early 1800’s) concerning use of the chestnut pod as a watercraft which implies possible movement of *C. australe* seeds.

*A very long time ago a carpet snake and a black snake started out in a canoe, in time of flood, from the mouth of the Pine River. Marvellous as it may seem, their canoe was just a shell of the Moreton Bay chestnut ("mai") — probably a gigantic one!*

**1.6**. There are numerous ethnographic records of *C. australe* use by Aboriginal people throughout tropical northern Queensland prior to or during early European colonisation including: the seeds as a food source (after preparation) [19, 20, 56, 57], sometimes after storage below ground for several months [58]; the bark fibre for fish and animal traps [56], nets and baskets [20]; the wood for spear throwers [59]; the seed pods as toy boats [60]; and as a seasonal cue for jungle fowl hunting [61, 62]. Similarly although to a lesser extent, Aboriginal uses in northern NSW were documented. For example the seeds were used as food resource after preparation [26, 63]; and the white flesh of the seedpod was used as a human poison by people from the Lismore area [64].

**1.7**. Transcript of an interview by EE relating to a story told by Uncle Ron Heron (RH) about movement of black bean seeds from north Queensland to NNSW (recorded by EE).

RH – [black bean trees] originally grew in Queensland and then when the husband and wife came down the coast she noticed that the dilly bag was getting lighter and lighter and there were little holes in it and they finally worked their way out of the little holes. And when she come towards Lismore [Northern NSW], there were none left see! All along the tracks of them in NSW and that where they come from see.

EE – When was that? Long time or?

RH – oh back in the Dreamtimes see…yeah long time ago

EE – Did she have a name or anything?

RH – No no…just that couple you know? Husband and wife couple…mmm.

**References**

1. Norledge M. Aboriginal Legends from Eastern Australia: The Richmond-Mary River Region. Sydney: Reed Publishing; 1968.
2. Petrie CC. Tom Petrie's reminiscences of early Queensland (dating from 1837). Watson, Ferguson & Company; 1904.
3. Dixon RMW. Searching for aboriginal languages: Memoirs of a field worker. Cambridge: Cambridge University Press; 1983.
4. Pedley H. Aboriginal life in the rainforest. Queensland Department of Education; 1992.
5. Harris DR. In: Jones NB, Reynolds V, editors. Human Behaviour and Adaptation. Taylor & Francis; 1978. pp. 113-134.
6. Kamminga J. Australian aboriginal timber quick search. Australian Institute of Canberra. Aboriginal and Torres Strait Islander Studies (AIATSIS); 2002.
7. Kerkhove R, Davis L. In: Davis S, editor. Balance-Unbalance 2013 International Conference: Future nature, future cultures. Noosa Biosphere Limited & CQU; 2013. pp. 165-171.
8. Roberts J, Fisher CJ, Gibson R, Popp T. A Guide to Traditional Aboriginal Rainforest Plant Use, by Kuku Yalanji of the Mossman Gorge. Bamanga Bubu Ngadimunku; 1995.
9. Clarke PA. Australian Aboriginal ethnometeorology and seasonal calendars. History and Anthropology. 2009; 20: 79-106.
10. King WJ. Indigenous Plant Conservation, Guanaba Indigenous Protected Area, Queensland. Australasian Plant Conservation: Journal of the Australian Network for Plant Conservation. 2011; 19: 16.
11. Webb LJ. The Use of Plant Medicines and Poisons by Australian Aborigines. Mankind. 1969; 7: 137-146.
