# Supplementary material for: From Songlines to genomes: Prehistoric assisted migration of a rain forest tree by Australian Aboriginal people
Source: PLoS One. 2017 Nov 8;12(11):e0186663. doi: 10.1371/journal.pone.0186663 (PMC5695580; doi:10.1371/journal.pone.0186663)
Supplement: S2 Appendix — (DOCX) [file pone.0186663.s002.docx]

**S2 Appendix: Interview questions and anthropological evidence**

**Introductory questions:**

1. What is your name?
2. What is your age?
3. Where is your Country?
4. Who passed on traditional knowledge to you?

**For each study species ask the following questions:**

1. What do you call this species? (show printed photos of the species seeds/flowers/habit)
2. What is your personal connection with the species (e. g. totemic or food)
3. Do you know any traditional uses of plant parts (e.g. for food, medicinal, ceremony, utensils)
4. Do you know any language names associated with each study species?
5. Do you know any stories about these species?
6. Do you know any songs about this species?
7. Do you know where we can find this species?
8. Are there any significant sites for this species?
9. Does this species occur in any song lines?
10. Do you know if ancestral beings or people in the past moved this species (or its seeds) around?
11. Who told you these stories?
12. Is there anyone else we could talk to about these species or places?
