# Supplementary material for: From Songlines to genomes: Prehistoric assisted migration of a rain forest tree by Australian Aboriginal people
Source: PLoS One. 2017 Nov 8;12(11):e0186663. doi: 10.1371/journal.pone.0186663 (PMC5695580; doi:10.1371/journal.pone.0186663)
Supplement: S3 Appendix — (DOCX) [file pone.0186663.s003.docx]

**S3 Appendix: Linguistic data**

**Table A: Linguistic information on the word forms used for *Castanospermum australe* within the sequence of Aboriginal languages covering the distribution of the species.** Rows of the table are arranged in a geographical sequence from north to south (see Figure S1 for more precise location).

| **Subgroup** | **Language** | **Form** | **Comments** | **Source** |
| --- | --- | --- | --- | --- |
| Yidinyic | Yidiny | *junggurra(a)*  *wirrum* |  | Dixon [73] |
|  | Djabugay | *yiwurra* |  | Patz (unpublished observations) |
| Yalanjic | Gugu Yalanji | *baway* |  | Hershberger and Hershberger [74] |
| Dyirbalic | Ngadjan (dialect of Dyirbal) | *ganyjuu* |  | Ngadjonji online [70] |
|  | Dyirbal | *mirrany* |  | Dixon [71] |
|  | Warrgamay | *wanga* |  |  |
| Maric | Warungu | *ganyjurr* ‘bean tree’ | Possible loan from Ngadjan Dyirbal *ganyjuu*, or source of Dyirbal loan (direction unknown) | Tsunoda [72] |
| Waka-Kabi | Butchulla (Batyala) | *mia* |  | Bell [65] |
| Durubalic | Yagara | *mai* | General word for ‘food’ in many languages; could be semantic shift or error in source | ASEDA Vocabulary 0703 |
| Bandjalangic | Numinbah (Yugambeh variety) | *boggum* |  | Gresty [66] |
|  | Githabul | *bugam* |  | Sharpe [67] |
|  | Waalubal (Bandjalang) | *bugam* ‘beanball’ |  |  |
| Gumbainygiric | Gumbaynggir | *binyjaalga* |  | Morelli [68] |
|  | Yaygirr | *wiguuli* |  | Morelli [69] |

**Figure A: Distribution of Eastern Australian Aboriginal language subgroups.**

**
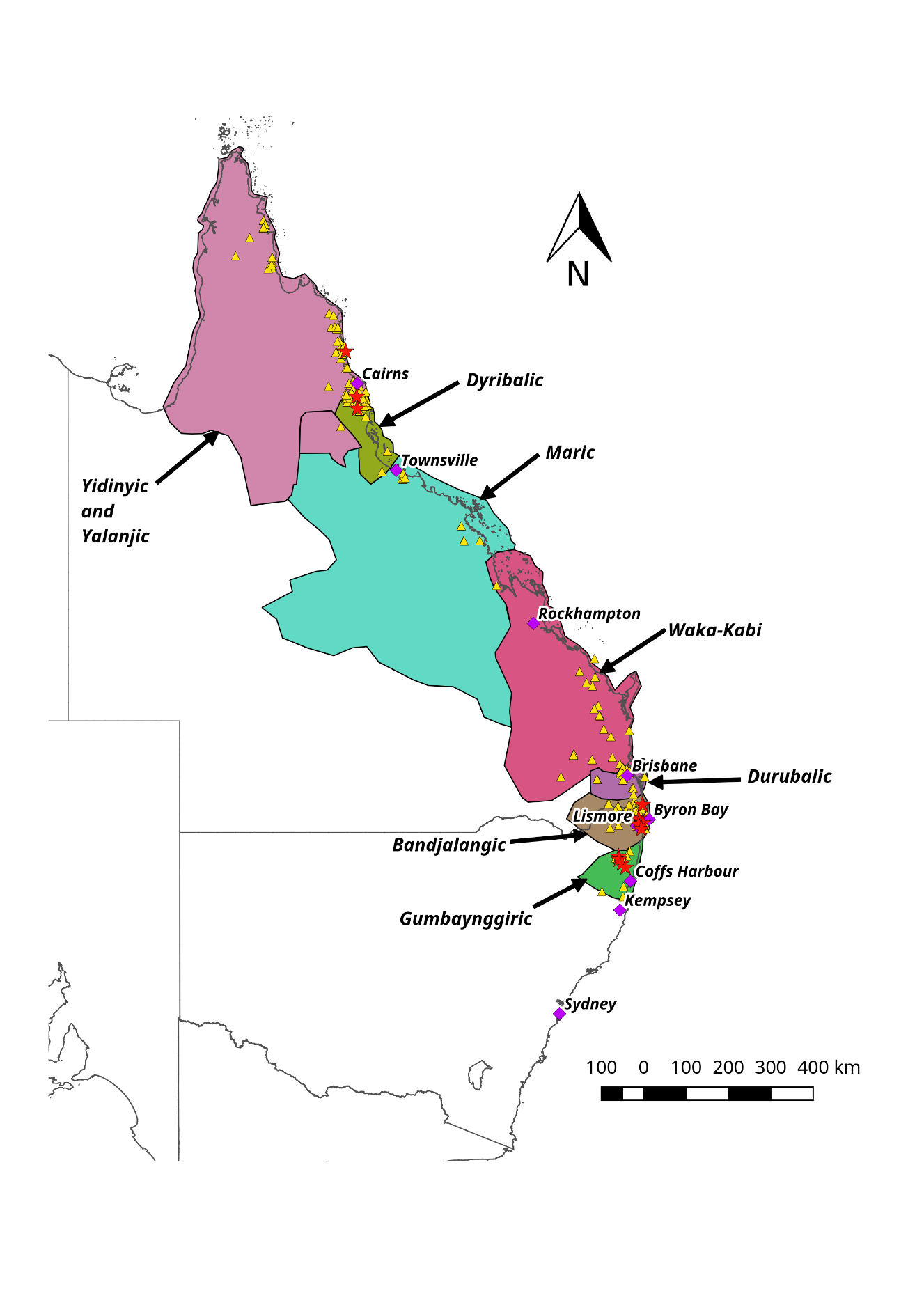
**

**References:**

1. Bell J. A sketch grammar of the Badjala language of Gari (Fraser Island). http://cat.lib.unimelb.edu.au/search~S6?/dGram+negative+bacteria./dgram+negative+bacteria/-3%2C-1%2C0%2CB/frameset&FF=dgrammar+butchulla+language&1%2C1%2C. Cited 16 May 2017.
2. Gresty JA. The Numinbah valley: its geography, history and Aboriginal associations. Queensland Geographical Journal. 1946; 51: 57-72.
3. Sharpe MC. All Bundjalung and Yugambeh Dictionary (Armidale, NSW) https://margaretcsharpe.wordpress.com/. 2013. Cited 28 March 2017.
4. Morelli S. Gumbaynggirr bijaarr jandaygam, ngaawa gugaarrigam: Gumbaynggirr dictionary and learner’s grammar, Muurrbay Aboriginal Language & Culture Co-operative. New South Wales: Nambucca Heads; 2008.
5. Morelli S. Yaygirr. Muurrbay Aboriginal Culture Centre; 2001.
6. Ngadjonji. Online vocabulary. http://www.earthsci.org/aboriginal/ngadjonji%20history/glossary/Ngadjon%20glossary.htm. Cited 28 March 2017.
7. Dixon RM. The Dyirbal language of north Queensland. Cambridge: Cambridge University Press; 1972.
8. Tsunoda T. A Grammar of Warrongo. Boston: Mouton Grammar Library 53 De Gruyter Mouton; 2011.
9. Dixon RMW, T. Irvine, Words of our country: stories, place names and vocabulary in Yidiny, the Aboriginal language of the Cairns-Yarrabah region. University of Queensland Press; 1991.
10. Hershberger HD, Hershberger R. Kuku-Yalanji Dictionary. Working papers of SIL-AAB, Series B Volume 7; 1986.
