# Supplementary material for: From Songlines to genomes: Prehistoric assisted migration of a rain forest tree by Australian Aboriginal people
Source: PLoS One. 2017 Nov 8;12(11):e0186663. doi: 10.1371/journal.pone.0186663 (PMC5695580; doi:10.1371/journal.pone.0186663)
Supplement: S4 Appendix — (DOCX) [file pone.0186663.s004.docx]

**S4 Appendix: Environmental Niche Models**

The climatic suitability of environments within the study area for *Castanospermum australe* was investigated using a species distribution model fitted using MaxEnt (version 3.3.3k; [75]). Climate data was 17 of the standard 19 bioclim variables (<http://fennerschool.anu.edu.au/research/products/anuclim-vrsn-61>); we excluded variables 8 and 9 because of the inherent discontinuities in values for these variables. The bioclim variables were computed using mean monthly minimum and maximum temperatures and mean monthly precipitation for the period 1983 to 2012. Data to compute these values was obtained from the eMAST repository (<http://dap.nci.org.au/thredds/catalog.html>).

Several topographic indices and soil structure data were also used as predictor variables in MaxEnt models. We used Topographic Wetness Index (TWI), Topographic Position Index (TPI), slope, aspect, and percent sand, silt and clay data downloaded from the CSIRO data access portal (<https://data.csiro.au/dap/home?execution=e1s1>). These data are provided at a very fine grid resolution which we re-sampled to match the eMAST climate data grid by computing the mean of values falling within an eMAST grid cell. Utility programs from the GDAL toolset (<http://www.gdal.org>) were used to perform the re-sampling.

Occurrence data for the model was obtained from the Atlas of Living Australia ([www.ala.org.au](http://www.ala.org.au/)) and filtered to include only herbarium-based species records, and to exclude specimens collected from municipal parks, street plantings and similar modern artificial records. Default parameter settings were used for MaxEnt except we used only linear, quadratic and product features to ensure smooth modeled relationships between occurrence density and each environmental variable [76, 77]. The fitted MaxEnt model was projected onto environmental variables representing likely conditions during the Last Glacial Maximum (LGM, c. 21ka BP) downscaled from Global Climate Model (GCM) runs produced for the Inter-governmental Panel on Climate Change Fifth Assessment Report [78].

LGM climate data for several GCMs which provided LGM runs, was downloaded from the Climate Model Intercomparison Project website (<https://esgf-node.llnl.gov/projects/esgf-llnl>). The GCMs included CNRM.CM5, COSMOS.ASO, FGOALS.g2, GISS.E2.R, IPSL.CM5A.LR, MPI.ESM.P and MRI.CGCM3. Two other GCMs provided LGM data (CCSM4 and MIROC.ESM) but were excluded after preliminary quality checks indicated that they produced anomalous estimated LGM climates. Monthly minimum and maximum temperatures and monthly precipitation data for LGM runs were downscaled using the delta method. In summary, the procedure involved: (a) Using pre-industrial control runs for each GCM to provide a baseline climate; (b) Calculating anomalies as the difference between each baseline and the corresponding LGM data; (c) Interpolating the anomalies onto the raster geometry of the eMAST climate data using bicubic splines; (d) Applying the interpolated anomalies to the observed monthly mean values provided by the eMAST data set; and (e) Computing the standard 19 bioclim variables.

Topographic and soil data were assumed not to have changed between Current (observed) conditions and the LGM.

**References**:

1. Phillips SJ, Anderson RP, Schapire RE. Maximum entropy modelling of species geographic distributions. Ecol. Model*.* 2006*;* 190: 231–259.
2. Austin MP. Continuum concept, ordination methods, and niche theory. Annu. Rev. Ecol. Syst. 1985; 39–61.
3. Austin M. Species distribution models and ecological theory: A critical assessment and some possible new approaches. Ecol. Model. 2007; 200**:** 1–19.
4. Core Writing Team, Pachauri RK, Meyer LA. Climate Change 2014: Synthesis Report. Contribution of Working Groups I, II and III to the Fifth Assessment Report of the Intergovernmental Panel on Climate Change. Geneva: IPCC; 2014. pp. 151.

**
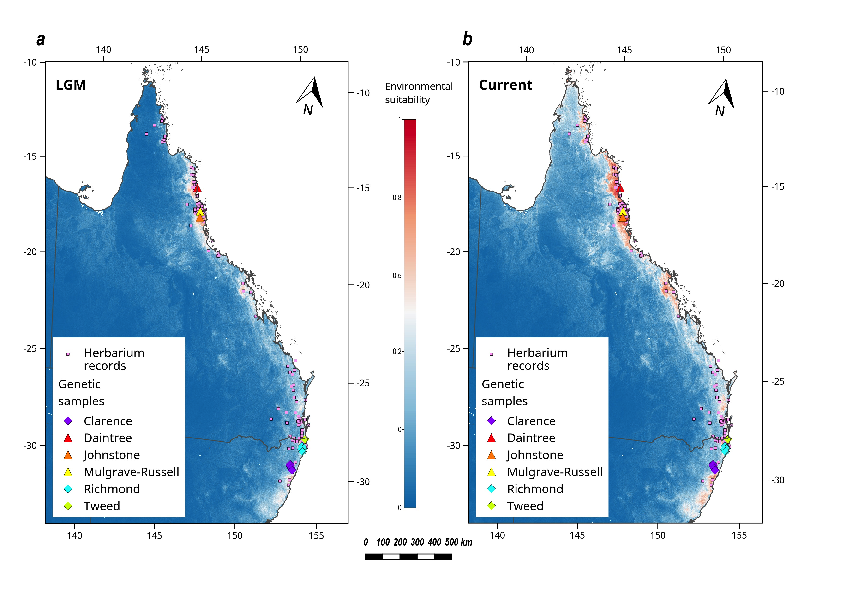
**

**Figure B: MaxEnt model of environmental suitability fitted to a) conditions representative of the Last Glacial Maximum (c. 21ka BP); b) current environmental conditions**. Scale for environmental suitability ranges from 0 (unsuitable) to 1 (highly suitable). The model indicates an increase in environmental suitability since the LGM across the entire range of the species.
