# Supplementary material for: From Songlines to genomes: Prehistoric assisted migration of a rain forest tree by Australian Aboriginal people
Source: PLoS One. 2017 Nov 8;12(11):e0186663. doi: 10.1371/journal.pone.0186663 (PMC5695580; doi:10.1371/journal.pone.0186663)
Supplement: S5 Appendix — (DOCX) [file pone.0186663.s005.docx]

**S5 Appendix: Additional genomic information**

The sequencing of 12 *Castanospermum australe* libraries generated 36.1Gbp of data or 0.24 billion quality reads, with an average of 21 million paired-end reads each library. The average read length after trimming was 140 bp, with an average Phred score of 36. The read mapping resulted in an average coverage of 1008x of the reference chloroplast genome (124,678bp) and an average of 4.17% of the total number of reads. For nuclear DNA, there was an average coverage of 3404x of the reference nuclear ribosomal DNA (5,813bp), and 0.6% of the total number of reads were mapped to the reference.

A *de novo* assembly DNA was performed on CLC using four *C. australe* libraries (RAM, VP, OW and OG) based on default settings. A total of 55,274,896 reads from these libraries generated 197,569 contigs, with an average of 1,387bp, and an N50 of 2,376. To identify chloroplast and nuclear sequences, 185 sequences consisting of known chloroplast genomes and nuclear ribosomal sequences were downloaded and a BLAST search for contigs of interest was conducted. This resulted with 16 chloroplast contigs and one nuclear contig with zero E-value. For the chloroplast contigs, the average length of the contigs was 8,041bp, with the longest contig at 31,079bp. From these four libraries, a total of 3,863,588 chloroplast reads were mapped to these contigs, and the average coverage was 3712x. There was a total of 892,791 nuclear reads mapped to the nuclear ribosomal contig (8,360bp) and the average coverage was 14,723x.

**Table B**: **Sequencing data summary for each *Castanospermum australe* population**.

| Population | CTBH | CF | GCK | HS | BS | Booy | BH | VP | RAM | OG | CC | OW |
| --- | --- | --- | --- | --- | --- | --- | --- | --- | --- | --- | --- | --- |
| No. of Quality Reads | 37281504 | 13407444 | 16021504 | 13717794 | 12923226 | 4294146 | 19266828 | 18208748 | 19490716 | 31300562 | 28228692 | 30028754 |
| No. of Gbp | 5.1 | 1.8 | 2.2 | 1.8 | 1.7 | 2.4 | 2.6 | 3.9 | 2.6 | 4.2 | 3.8 | 4 |
|  |  |  |  |  |  |  |  |  |  |  |  |  |
| **Chloroplast** |  |  |  |  |  |  |  |  |  |  |  |  |
| # Reads Mapped | 1060620 | 303777 | 449164 | 321898 | 411878 | 202532 | 417423 | 1209128 | 937755 | 3070178 | 1288480 | 1167579 |
| % Mapped | 2.84 | 2.27 | 2.80 | 2.35 | 3.19 | 4.72 | 2.17 | 6.64 | 4.81 | 9.81 | 4.56 | 3.89 |
| Avg. mapped read lengh | 146.98 | 146.64 | 146.76 | 144.4 | 146.22 | 272.71 | 146.89 | 150.99 | 146.95 | 147.23 | 147.03 | 147.13 |
| Avg. Coverage | 1179.69 | 328.62 | 499.68 | 340.45 | 446.31 | 385.47 | 455.3 | 1357.6 | 1017.22 | 3386.2 | 1422.39 | 1287.07 |
| Max Coverage | 3471 | 1153 | 3788 | 6274 | 1449 | 1701 | 1716 | 4130 | 4398 | 10847 | 4366 | 3718 |
| Reference length (bp) | 124678 | 124678 | 124678 | 124678 | 124678 | 124678 | 124678 | 124678 | 124678 | 124678 | 124678 | 124678 |
| Average Phred | 37 | 37 | 36 | 37 | 37 | 35 | 37 | 37 | 37 | 37 | 37 | 37 |
|  |  |  |  |  |  |  |  |  |  |  |  |  |
| **Nuclear ITS** |  |  |  |  |  |  |  |  |  |  |  |  |
| # Reads Mapped | 295210 | 78350 | 102976 | 58632 | 88846 | 18032 | 139186 | 134066 | 86286 | 193056 | 174862 | 175560 |
| % Mapped | 0.79 | 0.58 | 0.64 | 0.43 | 0.69 | 0.42 | 0.72 | 0.74 | 0.44 | 0.62 | 0.62 | 0.58 |
| Avg. mapped read lengh | 141.3 | 139.68 | 141.7 | 138.44 | 138.81 | 256.6 | 140.41 | 139.39 | 140.75 | 140.89 | 140.98 | 140.97 |
| Avg. Coverage | 7738.22 | 2059.02 | 2675.86 | 1528.77 | 2295.59 | 972.85 | 3664.65 | 3506.33 | 2257.85 | 5091.9 | 4543.29 | 4525.26 |
| Min Coverage | 6098 | 1717 | 2096 | 1023 | 1506 | 483 | 2538 | 2275 | 1509 | 3284 | 3067 | 3219 |
| Max Coverage | 11139 | 3008 | 3877 | 2442 | 3405 | 1232 | 5616 | 5078 | 3279 | 7494 | 6835 | 6674 |
| Reference length (bp) | 5813 | 5813 | 5813 | 5813 | 5813 | 5813 | 5813 | 5813 | 5813 | 5813 | 5813 | 5813 |
| Average Phred | 36 | 36 | 36 | 36 | 36 | 35 | 36 | 36 | 36 | 36 | 36 | 36 |
